# Crystal structure of di­aqua­(μ_2_-tri­ethyl­ene­tetra­minehexa­acetato)­dizinc tetra­hydrate

**DOI:** 10.1107/S2056989015002108

**Published:** 2015-02-07

**Authors:** Huan Liu, Li-Ping Lu

**Affiliations:** a Institute of Molecular Science, Key Laboratory of Chemical Biology and Molecular Engineering of the Education Ministry, Shanxi University, Taiyuan, Shanxi 030006, People’s Republic of China

**Keywords:** Crystal structure, binuclear Zn^II^ complex, tri­ethyl­ene­tetra­minehexa­acetic acid, crystal structure

## Abstract

The reaction of ZnO and tri­ethyl­ene­tetra­minehexaacetic acid (H_6_TTHA) in aqueous solution after refluxing yields the binuclear title compound, [Zn_2_(C_18_H_26_N_4_O_12_)(H_2_O)_2_]·4H_2_O. There is a centre of symmetry in the [Zn_2_(H_2_TTHA)(H_2_O)_2_] mol­ecule in the crystalline state. Both Zn^II^ ions are octahedrally surrounded and bound by an N_2_O_3_ donor set from the H_2_TTHA^4−^ anion and a water mol­ecule; the N atoms are *cis* and the water mol­ecule is *trans* to an N atom. The Zn⋯Zn separation is 7.562 (1) Å. An intra­molecular C—H⋯O inter­action is observed and both carboxyl­ate H atoms are disordered over two adjacent sites. In the crystal, the components are linked by O—H⋯O and C—H⋯O hydrogen bonds generating a three-dimensonal network.

## Related literature   

For general background to the complexes of tri­ethyl­ene­tetra­minehexaacetic acid, see: Long *et al.* (2003 ▸); Lu & Zhu (2014 ▸); Mondry & Starynowicz (1998 ▸); Ouyang *et al.* (2007 ▸); Sethi *et al.* (2012 ▸); Shi *et al.* (2006 ▸); Song *et al.* (2003 ▸); Thompson *et al.* (1998 ▸); Wang *et al.* (2003 ▸); Wullens *et al.* (1996 ▸). For related structures, see: Carlson *et al.* (2010 ▸); Qian *et al.* (2013 ▸).
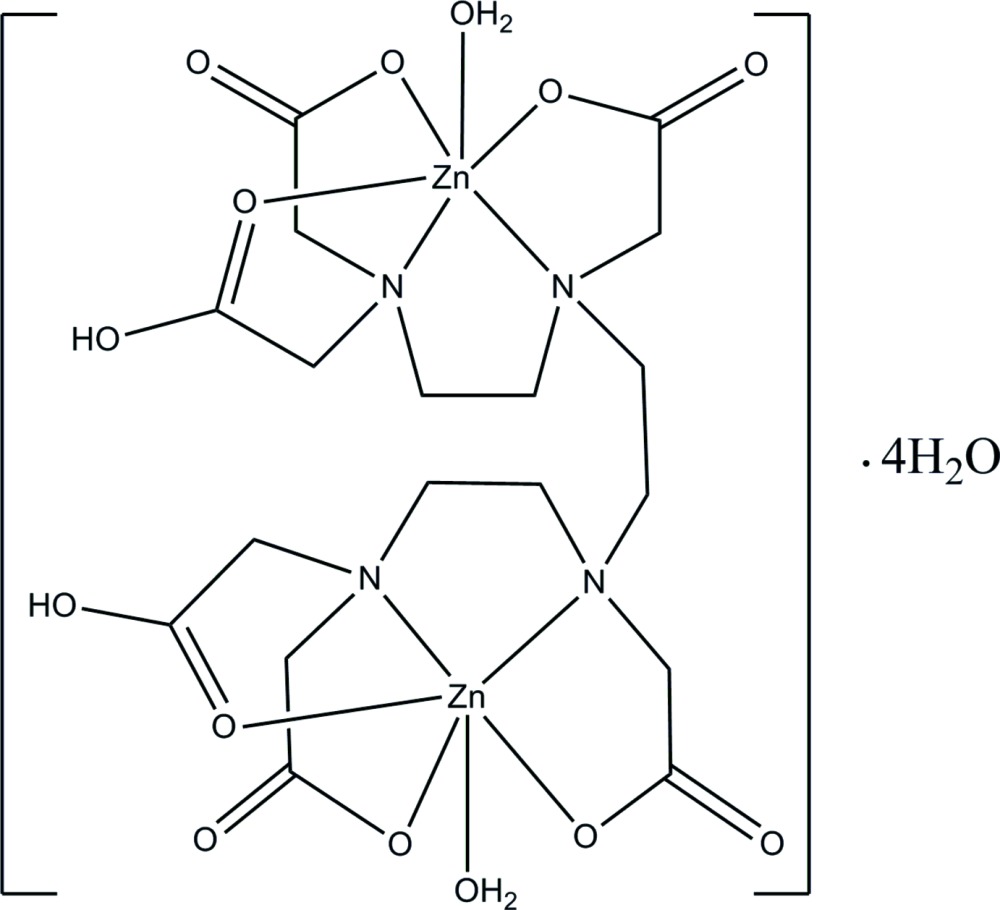


## Experimental   

### Crystal data  

[Zn_2_(C_18_H_26_N_4_O_12_)(H_2_O)_2_]·4H_2_O
*M* *_r_* = 729.26
Triclinic, 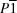

*a* = 7.1330 (14) Å
*b* = 8.7013 (16) Å
*c* = 11.979 (2) Å
α = 103.969 (2)°
β = 101.052 (2)°
γ = 100.882 (3)°
*V* = 686.2 (2) Å^3^
*Z* = 1
Mo *K*α radiation
μ = 1.84 mm^−1^
*T* = 298 K
0.28 × 0.22 × 0.20 mm

### Data collection  

Bruker SMART APEX CCD diffractometer
Absorption correction: multi-scan (*SADABS*; Bruker, 2000 ▸) *T*_min_ = 0.627, *T*_max_ = 0.710
3582 measured reflections
2384 independent reflections
2080 reflections with *I* > 2σ(*I*)
*R*_int_ = 0.057

### Refinement  

*R*[*F* ^2^ > 2σ(*F* ^2^)] = 0.034
*wR*(*F* ^2^) = 0.089
*S* = 0.99
2384 reflections
190 parameters
H-atom parameters constrained
Δρ_max_ = 0.49 e Å^−3^
Δρ_min_ = −0.76 e Å^−3^

### 

Data collection: *SMART* (Bruker, 2000 ▸); cell refinement: *SAINT* (Bruker, 2000 ▸); data reduction: *SAINT*; program(s) used to solve structure: *SHELXS97* (Sheldrick, 2008 ▸); program(s) used to refine structure: *SHELXL97* (Sheldrick, 2008 ▸); molecular graphics: *SHELXTL/PC* (Sheldrick, 2008 ▸); software used to prepare material for publication: *SHELXTL/PC*.

## Supplementary Material

Crystal structure: contains datablock(s) I. DOI: 10.1107/S2056989015002108/hb7351sup1.cif

Structure factors: contains datablock(s) I. DOI: 10.1107/S2056989015002108/hb7351Isup2.hkl

**Fig. 1.**
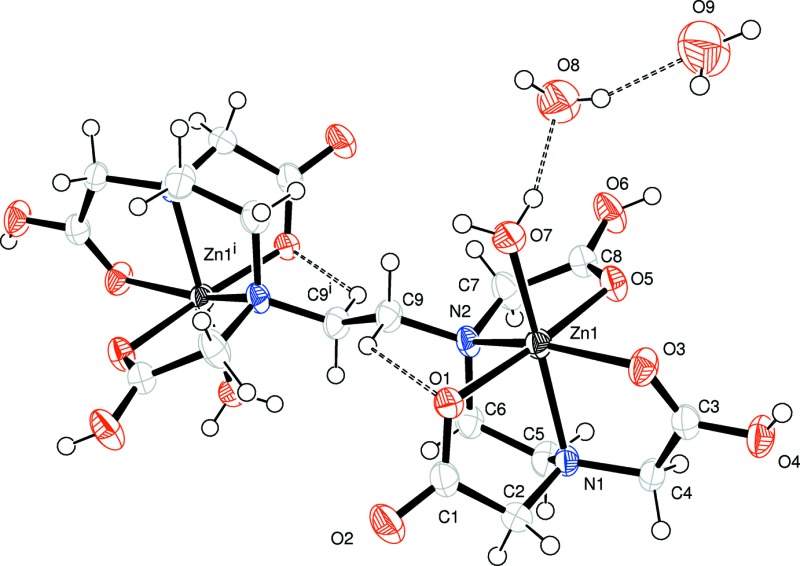
A view of the structure of the title complex with displacement ellipsoids drawn at the 50% probability level. Dash open line indicates hydrogen bonding interaction.

. DOI: 10.1107/S2056989015002108/hb7351fig1.tif
A view of the structure of the title complex with displacement ellipsoids drawn at the 50% probability level. Dash open line indicates hydrogen bonding inter­action.

**Fig. 2.**
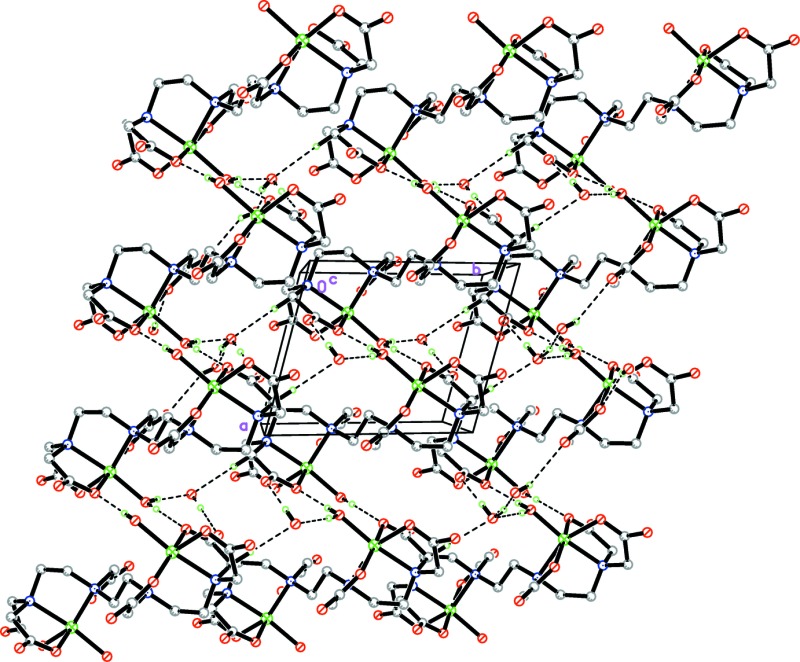
The packing diagram of the title compound, Zn dark green C gray, N blue,H light green, O red.

. DOI: 10.1107/S2056989015002108/hb7351fig2.tif
The packing diagram of the title compound, Zn dark green C gray, N blue,H light green, O red.

CCDC reference: 1046672

Additional supporting information:  crystallographic information; 3D view; checkCIF report

## Figures and Tables

**Table 1:** Selected bond lengths ()

Zn1O7	2.003(2)
Zn1O1	2.063(2)
Zn1O3	2.112(2)
Zn1O5	2.130(2)
Zn1N1	2.150(2)
Zn1N2	2.243(2)

**Table 2:** Hydrogen-bond geometry (, )

*D*H*A*	*D*H	H*A*	*D* *A*	*D*H*A*
C9H9*B*O1	0.97	2.54	3.198(4)	125
C2H2*A*O8^i^	0.97	2.52	3.476(4)	168
C5H5*B*O4^ii^	0.97	2.48	3.428(4)	166
O7H71O1^iii^	0.82	1.91	2.720(3)	169
O7H72O8	0.82	1.83	2.627(3)	164
O8H81O2^iii^	0.82	1.94	2.747(4)	166
O8H82O9	0.82	1.96	2.734(4)	157
O9H91O5^iv^	0.82	2.33	3.074(4)	151
O9H92O6^v^	0.82	2.33	3.033(4)	144

Symmetry codes: (i) 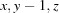; (ii) 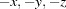; (iii) 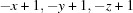; (iv) 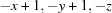; (v) 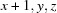.

## supplementary crystallographic information

### S1. Introduction

Tri­ethyl­ene­tetra­mine­hexa­acetic acid(H_6_TTHA), a multidentate ligand having ten potential coordinating sites (six oxygen atoms and four nitro­gen atoms), can play an important role in the self-assembly of chelating metals. It can be employed as a structure-directing agent to form main group metal complexes(Wullens *et al.*, 1996, Thompson *et al.*, 1998), transition-metal complexes(Song *et al.*, 2003, Long *et al.*, 2003, Qian *et al.*, 2013, Carlson *et al.*, 2010, Sethi *et al.*, 2012), lanthanide complexes(Wang *et al.*, 2003, Mondry & Starynowicz, 1998) and 3d–4f coordination polymers(Ouyang *et al.*, 2007, Shi *et al.*, 2006). To our knowledge, Zn(II) ions strongly inhibits many protein tyrosine phosphatases(Lu & Zhu, 2014). As part of the ongoing study of metal complexes inhibiting protein tyrosine phosphatases, the aim of us is to synthesize new zinc complexes employing polyamino polycarb­oxy­lic acids to form stable and soluble complexes. In this contribution, crystal structure of a binuclear zinc(II) complex of H_2_TTHA is reported.

### S2.1. Synthesis and crystallization

All chemicals were of reagent grade, commercially available and used without further purification. A mixture of H_6_TTHA (0.050 g, 0.10 mmol) and ZnO (0.016 g, 0.20 mmol) in 50 mL of deionized water in a flask was refluxed for 6 h. The clear solution was cooled to room temperature and filtered. The colorless filtrate was set aside at room temperature for three weeks. The title complex crystal was obtained as colourless blocks with yield 45%. Elemental Analysis(%): Cald. C 29.73, H 4.99, N 7.70; found C 29.52, H 5.05, N 7.57. Selected IR(KBr, cm^-1^): ν(O—H) 3445s, ν(C=O) 1732s, δ(O—H) 897m.

### S2.2. Refinement

Crystal data, data collection and structure refinement details are summarized in Table 2. H atoms attached to C of the title complex were placed in geometrically idealized positions with Csp^3^—H = 0.97Å and with Uiso(H) = 1.2Ueq(C). The carboxyl H4 and H6 atoms are each located close to a crystallographic inversion centre between pairs of symmetry equivalent atoms of O4 and O6. Both H atoms were thus refined as 50% occupied. The O—H distances were constrained to be 0.82 Å and Uiso= 1.5Ueq(O). H atoms attached to O(water) atoms were located from difference Fourier maps; their bond lengths were idealized to 0.82 Å and they were refined using a riding model, with Uiso(H) = 1.5Ueq(O).

### S3. Results and discussion

The molecular structure and the crystal packing are depicted in Figures 1 and 2, respectively. Selected bond lengths and bond angles are listed in Table 1. The Zn(II) ion has a six-coordinate pseudo-o­cta­hedral environment with two N and three O atoms from ligand H_2_TTHA anion as well as one water molecule. The complete binuclear molecule exists a centre of symmetry locating on the midpoint of bond C9—C9^i^ (Symmetry code i -x, 2-y, 2-z). The distance of both Zn(II) ions in the complex is 7.562 (1) Å. The distance of Zn—O(water) is 2.003 (2) Å, which is the shortest in all coordinate bonds of the title complex, while Zn—O bond lengths are in the range of 2.063 (2) to 2.130 (2) Å, and the Zn—N bond lengths are 2.150 (3) and 2.243 (2) Å, respectively. All the geometrical features compare very well with those in some similar structures, such as [Zn(H_2_O)_6_][Zn_2_(H_2_O)_2_(TTHA)].4H_2_O(Carlson *et al.*, 2010), [Co_2_(H_2_TTHA)(H_2_O)_2_].4H_2_O(Qian *et al.*, 2013).

In the structure of the title complex, numerous inter­molecular hydrogen bonds (O—H···O; Table 3) play an important role in stabilizing the structure and linking ions and solvent water molecules. Additional nonclassical C—H···O hydrogen bonds (Table 3) occur in the structure, with C—H···O angles in the range 125–168° and C···O distances between 3.198 (4) and 3.476 (4) Å.

### Crystal data

**Table:**

[Zn_2_(C_18_H_26_N_4_O_12_)(H_2_O)_2_]·4H_2_O	*Z* = 1
*M**_r_* = 729.26	*F*(000) = 378
Triclinic, *P*1	*D*_x_ = 1.765 Mg m^−^^3^
Hall symbol: -P 1	Mo *K*α radiation, λ = 0.71073 Å
*a* = 7.1330 (14) Å	Cell parameters from 2008 reflections
*b* = 8.7013 (16) Å	θ = 2.6–26.9°
*c* = 11.979 (2) Å	µ = 1.84 mm^−^^1^
α = 103.969 (2)°	*T* = 298 K
β = 101.052 (2)°	Block, colorless
γ = 100.882 (3)°	0.28 × 0.22 × 0.20 mm
*V* = 686.2 (2) Å^3^	

### Data collection

**Table:**

Bruker SMART APEX CCD diffractometer	2384 independent reflections
Radiation source: fine-focus sealed tube	2080 reflections with *I* > 2σ(*I*)
Graphite monochromator	*R*_int_ = 0.057
ω scans	θ_max_ = 25.0°, θ_min_ = 1.8°
Absorption correction: multi-scan (*SADABS*; Bruker, 2000)	*h* = −7→8
*T*_min_ = 0.627, *T*_max_ = 0.710	*k* = −10→10
3582 measured reflections	*l* = −14→9

### Refinement

**Table:**

Refinement on *F*^2^	Primary atom site location: structure-invariant direct methods
Least-squares matrix: full	Secondary atom site location: difference Fourier map
*R*[*F*^2^ > 2σ(*F*^2^)] = 0.034	Hydrogen site location: inferred from neighbouring sites
*wR*(*F*^2^) = 0.089	H-atom parameters constrained
*S* = 0.99	*w* = 1/[σ^2^(*F*_o_^2^) + (0.0505*P*)^2^] where *P* = (*F*_o_^2^ + 2*F*_c_^2^)/3
2384 reflections	(Δ/σ)_max_ < 0.001
190 parameters	Δρ_max_ = 0.49 e Å^−^^3^
0 restraints	Δρ_min_ = −0.75 e Å^−^^3^

### Special details

**Table:**

Geometry. All e.s.d.'s (except the e.s.d. in the dihedral angle between two l.s. planes) are estimated using the full covariance matrix. The cell e.s.d.'s are taken into account individually in the estimation of e.s.d.'s in distances, angles and torsion angles; correlations between e.s.d.'s in cell parameters are only used when they are defined by crystal symmetry. An approximate (isotropic) treatment of cell e.s.d.'s is used for estimating e.s.d.'s involving l.s. planes.
Refinement. Refinement of *F*^2^ against all reflections. The weighted *R*-factor *wR* and goodness of fit *S* are based on *F*^2^, conventional *R*-factors *R* are based on *F*, with *F* set to zero for negative *F*^2^. The threshold expression of *F*^2^ > σ(*F*^2^) is used only for calculating *R*-factors(gt) *etc*. and is not relevant to the choice of reflections for refinement. *R*-factors based on *F*^2^ are statistically about twice as large as those based on *F*, and *R*- factors based on all data will be even larger.

### Fractional atomic coordinates and isotropic or equivalent isotropic displacement parameters (Å^2^)

**Table:**

	*x*	*y*	*z*	*U*_iso_*/*U*_eq_	Occ. (<1)
Zn1	0.27323 (5)	0.29051 (4)	0.29273 (3)	0.02494 (14)	
N1	0.0979 (4)	0.0459 (3)	0.2561 (2)	0.0249 (5)	
N2	0.0131 (4)	0.3615 (3)	0.3509 (2)	0.0257 (5)	
O1	0.3853 (3)	0.2527 (2)	0.45320 (18)	0.0296 (5)	
O2	0.3234 (4)	0.0827 (3)	0.5614 (2)	0.0516 (7)	
O3	0.4168 (3)	0.1580 (3)	0.17890 (19)	0.0331 (5)	
O4	0.3493 (3)	−0.0649 (3)	0.0251 (2)	0.0390 (6)	
H4	0.4574	−0.0369	0.0120	0.058*	0.50
O5	0.1248 (3)	0.3442 (3)	0.14014 (18)	0.0312 (5)	
O6	−0.0748 (4)	0.4953 (3)	0.0850 (2)	0.0437 (6)	
H6	−0.0393	0.4897	0.0232	0.065*	0.50
O7	0.4665 (3)	0.5080 (2)	0.3369 (2)	0.0377 (5)	
H71	0.5033	0.5712	0.4042	0.057*	
H72	0.4553	0.5622	0.2898	0.057*	
C1	0.3041 (5)	0.1170 (4)	0.4670 (3)	0.0315 (7)	
C2	0.1742 (5)	−0.0156 (4)	0.3559 (3)	0.0332 (7)	
H2A	0.2499	−0.0926	0.3294	0.040*	
H2B	0.0634	−0.0747	0.3772	0.040*	
C3	0.3124 (4)	0.0213 (4)	0.1162 (3)	0.0293 (7)	
C4	0.1217 (5)	−0.0520 (4)	0.1428 (3)	0.0300 (7)	
H4A	0.0114	−0.0583	0.0789	0.036*	
H4B	0.1204	−0.1623	0.1467	0.036*	
C5	−0.1051 (4)	0.0634 (4)	0.2504 (3)	0.0341 (7)	
H5A	−0.1871	−0.0370	0.2539	0.041*	
H5B	−0.1585	0.0825	0.1756	0.041*	
C6	−0.1089 (4)	0.2049 (4)	0.3529 (3)	0.0308 (7)	
H6A	−0.2438	0.2133	0.3478	0.037*	
H6B	−0.0602	0.1835	0.4275	0.037*	
C7	−0.0878 (5)	0.4310 (4)	0.2619 (3)	0.0383 (8)	
H7A	−0.0791	0.5449	0.2996	0.046*	
H7B	−0.2262	0.3740	0.2356	0.046*	
C8	−0.0028 (5)	0.4194 (4)	0.1545 (3)	0.0303 (7)	
C9	0.0821 (4)	0.4801 (4)	0.4711 (3)	0.0293 (7)	
H9A	0.1606	0.5808	0.4657	0.035*	
H9B	0.1671	0.4367	0.5221	0.035*	
O8	0.4369 (4)	0.7275 (3)	0.2220 (2)	0.0575 (7)	
H81	0.5196	0.7918	0.2798	0.086*	
H82	0.4963	0.6924	0.1731	0.086*	
O9	0.6210 (6)	0.6889 (4)	0.0405 (3)	0.0923 (12)	
H91	0.6512	0.6831	−0.0229	0.138*	
H92	0.6638	0.6205	0.0673	0.138*	

### Atomic displacement parameters (Å^2^)

**Table:**

	*U*^11^	*U*^22^	*U*^33^	*U*^12^	*U*^13^	*U*^23^
Zn1	0.0260 (2)	0.0262 (2)	0.0228 (2)	0.00595 (14)	0.00896 (14)	0.00540 (14)
N1	0.0269 (13)	0.0268 (12)	0.0197 (13)	0.0050 (10)	0.0083 (10)	0.0037 (10)
N2	0.0287 (14)	0.0320 (13)	0.0212 (13)	0.0122 (11)	0.0122 (11)	0.0081 (11)
O1	0.0362 (12)	0.0283 (11)	0.0223 (11)	0.0051 (9)	0.0041 (9)	0.0079 (9)
O2	0.0700 (18)	0.0531 (15)	0.0268 (13)	0.0008 (13)	0.0058 (12)	0.0186 (12)
O3	0.0289 (12)	0.0353 (12)	0.0319 (13)	0.0060 (10)	0.0140 (10)	0.0003 (10)
O4	0.0419 (14)	0.0420 (13)	0.0306 (13)	0.0098 (11)	0.0181 (10)	−0.0007 (11)
O5	0.0339 (12)	0.0416 (12)	0.0242 (11)	0.0155 (10)	0.0122 (9)	0.0121 (10)
O6	0.0534 (16)	0.0635 (16)	0.0340 (13)	0.0340 (13)	0.0195 (12)	0.0288 (12)
O7	0.0442 (14)	0.0321 (12)	0.0300 (13)	−0.0023 (10)	0.0068 (10)	0.0075 (10)
C1	0.0356 (18)	0.0370 (17)	0.0258 (17)	0.0140 (15)	0.0111 (14)	0.0097 (14)
C2	0.0449 (19)	0.0280 (16)	0.0296 (18)	0.0088 (14)	0.0127 (15)	0.0111 (14)
C3	0.0320 (17)	0.0348 (17)	0.0245 (17)	0.0140 (14)	0.0092 (14)	0.0086 (14)
C4	0.0348 (18)	0.0285 (15)	0.0231 (16)	0.0047 (13)	0.0093 (13)	0.0017 (13)
C5	0.0263 (17)	0.0383 (18)	0.0325 (18)	0.0018 (14)	0.0097 (14)	0.0038 (15)
C6	0.0256 (16)	0.0378 (17)	0.0302 (18)	0.0066 (14)	0.0136 (13)	0.0074 (14)
C7	0.043 (2)	0.055 (2)	0.0338 (19)	0.0285 (17)	0.0212 (16)	0.0214 (17)
C8	0.0318 (17)	0.0341 (17)	0.0258 (17)	0.0094 (14)	0.0080 (13)	0.0088 (14)
C9	0.0277 (16)	0.0328 (16)	0.0285 (17)	0.0088 (14)	0.0136 (13)	0.0048 (13)
O8	0.0607 (18)	0.0574 (17)	0.0524 (17)	0.0100 (14)	0.0120 (14)	0.0173 (14)
O9	0.115 (3)	0.089 (2)	0.108 (3)	0.047 (2)	0.076 (3)	0.036 (2)

### Geometric parameters (Å, º)

**Table:**

Zn1—O7	2.003 (2)	C1—C2	1.530 (4)
Zn1—O1	2.063 (2)	C2—H2A	0.9700
Zn1—O3	2.112 (2)	C2—H2B	0.9700
Zn1—O5	2.130 (2)	C3—C4	1.516 (4)
Zn1—N1	2.150 (2)	C4—H4A	0.9700
Zn1—N2	2.243 (2)	C4—H4B	0.9700
N1—C5	1.474 (4)	C5—C6	1.523 (4)
N1—C4	1.477 (4)	C5—H5A	0.9700
N1—C2	1.477 (4)	C5—H5B	0.9700
N2—C6	1.480 (4)	C6—H6A	0.9700
N2—C7	1.483 (4)	C6—H6B	0.9700
N2—C9	1.483 (4)	C7—C8	1.514 (4)
O1—C1	1.277 (4)	C7—H7A	0.9700
O2—C1	1.227 (4)	C7—H7B	0.9700
O3—C3	1.246 (4)	C9—C9^i^	1.523 (5)
O4—C3	1.270 (4)	C9—H9A	0.9700
O4—H4	0.8200	C9—H9B	0.9700
O5—C8	1.232 (4)	O8—H81	0.8203
O6—C8	1.274 (4)	O8—H82	0.8203
O6—H6	0.8199	O9—H91	0.8207
O7—H71	0.8199	O9—H92	0.8211
O7—H72	0.8200		
			
O7—Zn1—O1	91.79 (8)	N1—C2—H2B	108.6
O7—Zn1—O3	97.19 (9)	C1—C2—H2B	108.6
O1—Zn1—O3	102.29 (8)	H2A—C2—H2B	107.6
O7—Zn1—O5	88.93 (9)	O3—C3—O4	124.8 (3)
O1—Zn1—O5	171.03 (8)	O3—C3—C4	120.6 (3)
O3—Zn1—O5	86.48 (8)	O4—C3—C4	114.6 (3)
O7—Zn1—N1	172.37 (9)	N1—C4—C3	111.7 (2)
O1—Zn1—N1	82.46 (8)	N1—C4—H4A	109.3
O3—Zn1—N1	79.26 (9)	C3—C4—H4A	109.3
O5—Zn1—N1	97.53 (9)	N1—C4—H4B	109.3
O7—Zn1—N2	101.71 (9)	C3—C4—H4B	109.3
O1—Zn1—N2	92.61 (9)	H4A—C4—H4B	107.9
O3—Zn1—N2	155.51 (9)	N1—C5—C6	110.8 (2)
O5—Zn1—N2	78.50 (8)	N1—C5—H5A	109.5
N1—Zn1—N2	83.63 (9)	C6—C5—H5A	109.5
C5—N1—C4	113.2 (2)	N1—C5—H5B	109.5
C5—N1—C2	112.3 (2)	C6—C5—H5B	109.5
C4—N1—C2	111.4 (2)	H5A—C5—H5B	108.1
C5—N1—Zn1	104.87 (18)	N2—C6—C5	111.6 (2)
C4—N1—Zn1	107.68 (17)	N2—C6—H6A	109.3
C2—N1—Zn1	106.79 (18)	C5—C6—H6A	109.3
C6—N2—C7	112.4 (3)	N2—C6—H6B	109.3
C6—N2—C9	111.3 (2)	C5—C6—H6B	109.3
C7—N2—C9	112.1 (2)	H6A—C6—H6B	108.0
C6—N2—Zn1	103.30 (16)	N2—C7—C8	113.6 (2)
C7—N2—Zn1	108.16 (17)	N2—C7—H7A	108.8
C9—N2—Zn1	109.12 (17)	C8—C7—H7A	108.8
C1—O1—Zn1	115.50 (19)	N2—C7—H7B	108.8
C3—O3—Zn1	113.58 (19)	C8—C7—H7B	108.8
C3—O4—H4	118.3	H7A—C7—H7B	107.7
C8—O5—Zn1	116.02 (19)	O5—C8—O6	125.4 (3)
C8—O6—H6	118.2	O5—C8—C7	121.3 (3)
Zn1—O7—H71	123.8	O6—C8—C7	113.3 (3)
Zn1—O7—H72	117.0	N2—C9—C9^i^	114.6 (3)
H71—O7—H72	108.0	N2—C9—H9A	108.6
O2—C1—O1	125.6 (3)	C9^i^—C9—H9A	108.6
O2—C1—C2	117.1 (3)	N2—C9—H9B	108.6
O1—C1—C2	117.3 (3)	C9^i^—C9—H9B	108.6
N1—C2—C1	114.6 (2)	H9A—C9—H9B	107.6
N1—C2—H2A	108.6	H81—O8—H82	107.0
C1—C2—H2A	108.6	H91—O9—H92	106.8
			
O1—Zn1—N1—C5	111.87 (18)	O7—Zn1—O5—C8	−87.6 (2)
O3—Zn1—N1—C5	−144.02 (19)	O3—Zn1—O5—C8	175.2 (2)
O5—Zn1—N1—C5	−59.08 (19)	N1—Zn1—O5—C8	96.5 (2)
N2—Zn1—N1—C5	18.38 (18)	N2—Zn1—O5—C8	14.6 (2)
O1—Zn1—N1—C4	−127.26 (19)	Zn1—O1—C1—O2	−166.3 (3)
O3—Zn1—N1—C4	−23.16 (18)	Zn1—O1—C1—C2	14.7 (3)
O5—Zn1—N1—C4	61.78 (19)	C5—N1—C2—C1	−97.9 (3)
N2—Zn1—N1—C4	139.24 (19)	C4—N1—C2—C1	133.9 (3)
O1—Zn1—N1—C2	−7.52 (18)	Zn1—N1—C2—C1	16.6 (3)
O3—Zn1—N1—C2	96.59 (19)	O2—C1—C2—N1	159.0 (3)
O5—Zn1—N1—C2	−178.48 (18)	O1—C1—C2—N1	−21.9 (4)
N2—Zn1—N1—C2	−101.02 (19)	Zn1—O3—C3—O4	164.5 (2)
O7—Zn1—N2—C6	−164.05 (17)	Zn1—O3—C3—C4	−13.1 (4)
O1—Zn1—N2—C6	−71.68 (18)	C5—N1—C4—C3	138.9 (3)
O3—Zn1—N2—C6	56.2 (3)	C2—N1—C4—C3	−93.3 (3)
O5—Zn1—N2—C6	109.49 (18)	Zn1—N1—C4—C3	23.4 (3)
N1—Zn1—N2—C6	10.43 (18)	O3—C3—C4—N1	−7.9 (4)
O7—Zn1—N2—C7	76.6 (2)	O4—C3—C4—N1	174.3 (2)
O1—Zn1—N2—C7	169.0 (2)	C4—N1—C5—C6	−162.2 (3)
O3—Zn1—N2—C7	−63.1 (3)	C2—N1—C5—C6	70.6 (3)
O5—Zn1—N2—C7	−9.9 (2)	Zn1—N1—C5—C6	−45.0 (3)
N1—Zn1—N2—C7	−108.9 (2)	C7—N2—C6—C5	78.5 (3)
O7—Zn1—N2—C9	−45.53 (19)	C9—N2—C6—C5	−154.9 (3)
O1—Zn1—N2—C9	46.84 (19)	Zn1—N2—C6—C5	−37.9 (3)
O3—Zn1—N2—C9	174.73 (19)	N1—C5—C6—N2	59.6 (3)
O5—Zn1—N2—C9	−131.99 (19)	C6—N2—C7—C8	−107.8 (3)
N1—Zn1—N2—C9	128.95 (19)	C9—N2—C7—C8	126.0 (3)
O7—Zn1—O1—C1	−178.9 (2)	Zn1—N2—C7—C8	5.6 (3)
O3—Zn1—O1—C1	−81.1 (2)	Zn1—O5—C8—O6	162.7 (3)
N1—Zn1—O1—C1	−3.9 (2)	Zn1—O5—C8—C7	−16.4 (4)
N2—Zn1—O1—C1	79.3 (2)	N2—C7—C8—O5	6.7 (5)
O7—Zn1—O3—C3	−166.2 (2)	N2—C7—C8—O6	−172.5 (3)
O1—Zn1—O3—C3	100.4 (2)	C6—N2—C9—C9^i^	−58.1 (4)
O5—Zn1—O3—C3	−77.7 (2)	C7—N2—C9—C9^i^	68.7 (4)
N1—Zn1—O3—C3	20.6 (2)	Zn1—N2—C9—C9^i^	−171.5 (3)
N2—Zn1—O3—C3	−25.8 (3)		

Symmetry code: (i) −*x*, −*y*+1, −*z*+1.

### Hydrogen-bond geometry (Å, º)

**Table:**

*D*—H···*A*	*D*—H	H···*A*	*D*···*A*	*D*—H···*A*
C9—H9*B*···O1	0.97	2.54	3.198 (4)	125
C2—H2*A*···O8^ii^	0.97	2.52	3.476 (4)	168
C5—H5*B*···O4^iii^	0.97	2.48	3.428 (4)	166
O7—H71···O1^iv^	0.82	1.91	2.720 (3)	169
O7—H72···O8	0.82	1.83	2.627 (3)	164
O8—H81···O2^iv^	0.82	1.94	2.747 (4)	166
O8—H82···O9	0.82	1.96	2.734 (4)	157
O9—H91···O5^v^	0.82	2.33	3.074 (4)	151
O9—H92···O6^vi^	0.82	2.33	3.033 (4)	144

Symmetry codes: (ii) *x*, *y*−1, *z*; (iii) −*x*, −*y*, −*z*; (iv) −*x*+1, −*y*+1, −*z*+1; (v) −*x*+1, −*y*+1, −*z*; (vi) *x*+1, *y*, *z*.
