# Supplementary figures and images for: Crystal structure of di­aqua­(μ2-tri­ethyl­ene­tetra­minehexa­acetato)­dizinc tetra­hydrate
Source: Acta Crystallogr E Crystallogr Commun. 2015 Feb 7;71(Pt 3):m55–6. doi: 10.1107/S2056989015002108 (PMC4350722; doi:10.1107/S2056989015002108)

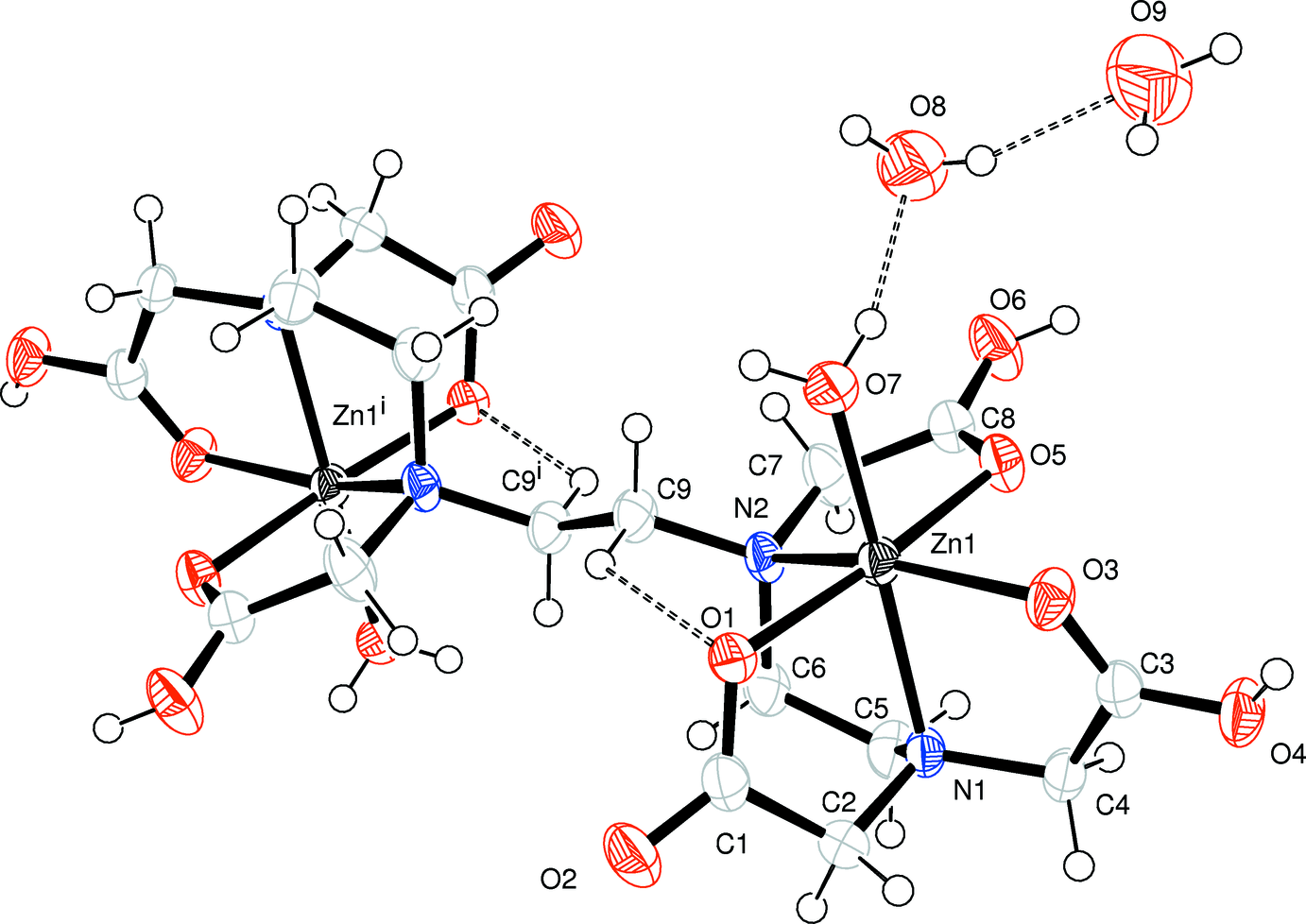

Supplement: Supplementary file 3 [file e-71-00m55-fig1.tif]

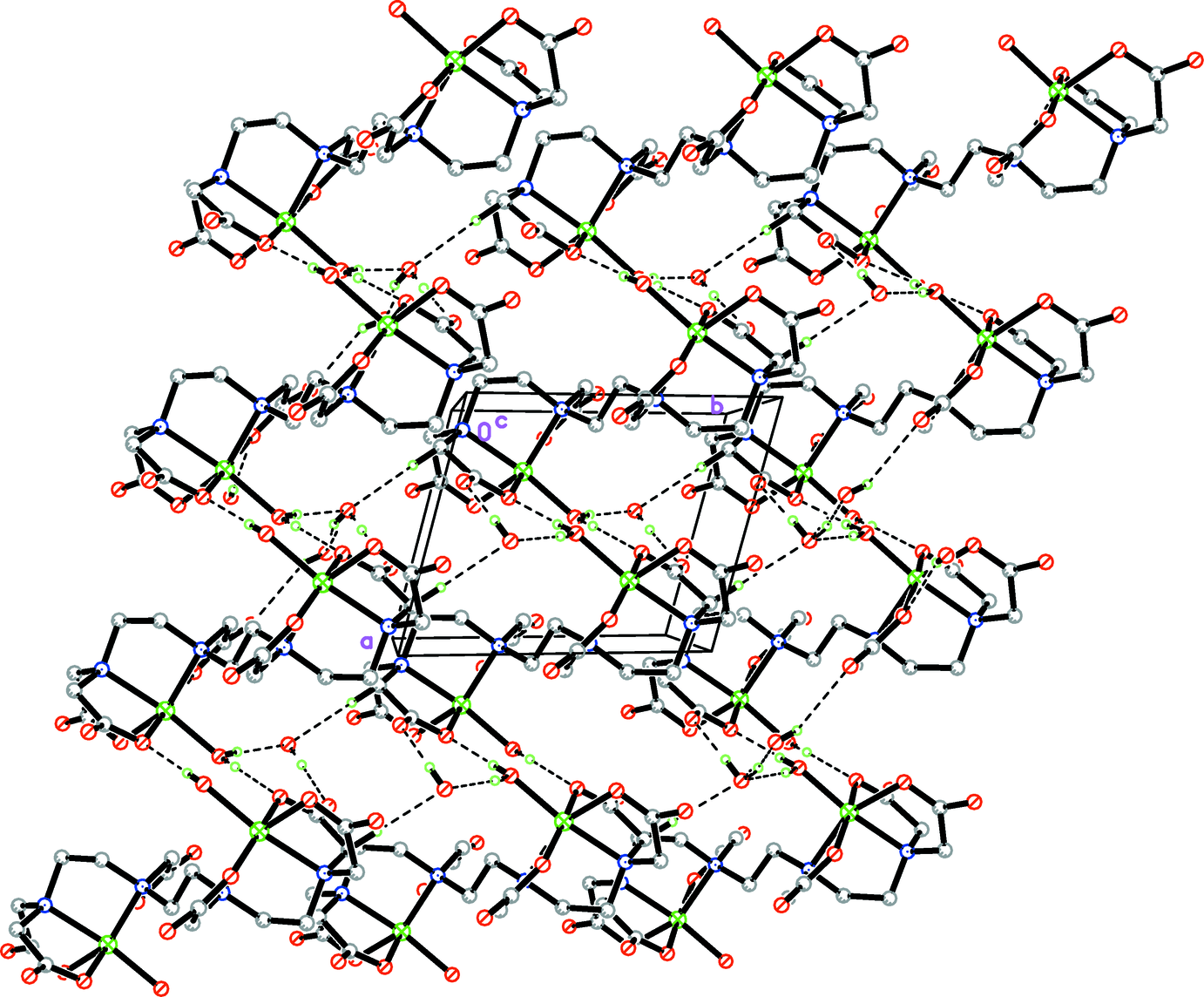

Supplement: Supplementary file 4 [file e-71-00m55-fig2.tif]
